# Phenological patterns of tropical mountain forest trees across the neotropics: evidence from herbarium specimens

**DOI:** 10.1098/rspb.2024.2748

**Published:** 2025-02-26

**Authors:** J. C. Ordoñez, C. Tovar, B. E. Walker, J. Wheeler, S. Ayala-Ruano, K. Aguirre-Carvajal, S. M. McMahon, F. Cuesta

**Affiliations:** ^1^ Grupo de Investigación en Biodiversidad, Medio Ambiente y Salud (BIOMAS), Universidad de Las Américas, Quito 170124, Ecuador; ^2^ Royal Botanic Gardens, Kew, Surrey TW9 3AB, UK; ^3^ School of Biological and Behavioural Sciences, Queen Mary University of London, 50 Under Knoll, Peasedown St John, Bath BA2 8TY, UK; ^4^ Faculty of Science and Engineering, Maastricht University, Maastricht 6200 MD, The Netherlands; ^5^ Multiomics Network Analytics Group, Novo Nordisk Foundation Center for Biosustainability (DTU Biosustain), Copenhagen, 2800 Kgs. Lyngby, Denmark; ^6^ Bio-Cheminformatics Research Group, Universidad de Las Américas, Quito 170124, Ecuador; ^7^ Smithsonian Environmental Research Center, Edgewater, MD 21037-0028, USA; ^8^ Forest Global Earth Observatory, Smithsonian Tropical Research Institute, Panama City Apartado Postal 0843-03092, Panama; ^9^ Global Solutions and Research Center, Universidad San Francisco de Quito, Quito, Ecuador

**Keywords:** montane forests, random forest, circular statistics, neotropics, vouchers, natural language processing

## Abstract

The flowering phenology of many tropical mountain forest tree species remains poorly understood, including flowering synchrony and its drivers across neotropical ecosystems. We obtained herbarium records for 427 tree species from a long-term monitoring transect on the northwestern Ecuadorian Andes, sourced from the Global Biodiversity Information Facility and the Herbario Nacional del Ecuador. Using machine learning algorithms, we identified flowering phenophases from digitized specimen labels and applied circular statistics to build phenological calendars across six climatic regions within the neotropics. We found 47 939 herbarium records, of which 14 938 were classified as flowering by Random Forest Models. We constructed phenological calendars for six regions and 86 species with at least 20 flowering records. Phenological patterns varied considerably across regions, among species within regions, and within species across regions. There was limited interannual synchronicity in flowering patterns within regions primarily driven by bimodal species whose flowering peaks coincided with irradiance peaks. The predominantly high variability of phenological patterns among species and within species likely confers adaptative advantages by reducing interspecific competition during reproductive periods and promoting species coexistence in highly diverse regions with little or no seasonality.

## 1. Introduction

Tropical montane forests (TMFs) develop at elevations above approximately 500 m a.s.l., reaching 3800 m a.s.l. on the outer slopes of the equatorial Andes [1]. These are among the most diverse ecosystems on the planet [2]. TMF conservation and restoration are high on the global conservation agenda, and many efforts are directed towards the restoration of TMF, which is focused on tree planting schemes or the management of forest natural regeneration [3]. Nevertheless, we know very little about the fundamental aspects of the reproductive biology of most tree species from TMFs, such as their flowering phenology. These patterns may be critical to understanding the mechanisms determining the current range of TMF species. Phenology offers insight into how species might shift their range under climate change based on the competition and synchrony of community reproduction patterns [4].

The few available studies of the phenology of flowering and fruiting of TMF species focus on a few species in a few locations [5,6]. The lack of biogeographical and ecological information about such a rich biome is partially due to the great diversity and high frequency of rare species in TMF [7], and the difficulty of collecting the data required to assess population dynamics, primarily through collections and field observations. In recent years, studies based on herbaria data, citizen science platforms and data mining in secondary sources have been used to examine phenology with robust results [8,9]. These studies focused on (i) reconstructing phenological phases (e.g. annual timing of flowering and fruiting) and assessing the triggers of phenological changes (environmental, biotic or phylogenetic; [8,10,11]), and recently, (ii) evaluating the potential impacts on flowering timing due to climate change [12–14]. Most studies using phenological data from herbarium specimens concentrate on temperate and Arctic ecosystems [9], and only a few are available from tropical regions [14–16], focusing on single genera or selected species within a family. To our knowledge, no study has assessed the phenology of a large pool of tree species in humid TMF in the Americas. Also, most methods that derive phenological status from herbarium records require a visual examination of the vouchers, but there is little use of the rich information registered in voucher labels. New tools based on machine learning algorithms could open interesting opportunities to access information from labels [16].

Temperature periodicity and photoperiod strongly constrain phenology in temperate zones [17], driving seasonal synchrony in reproduction, and as latitude decreases there is less synchronic flowering [18]. In tropical forests, reproductive phenological patterns vary in timing, frequency and duration [18–20]. When synchronic flowering occurs in the tropics, this suggests strong environmental filters may constrain reproductive processes, and species must adapt their phenology to fit within suitable seasons. Evidence indicates that the timing of flowering in some species is influenced by environmental drivers such as rainfall, particularly in regions with marked dry seasons, and irradiance [21–23] or daily insolation [24,25], even close to the Equator.

In Andean TMFs, the few published studies on phenological patterns associated with environmental drivers report mixed results, often focusing on a few species. For example, in dry sites, interspecific synchronicity in flowering time has been linked to precipitation and radiation, whereas in humid sites, both synchronous and asynchronous flowering patterns have been observed [6,26]. Thus, it remains unclear whether there is a consistent degree of flowering synchrony among species from TMFs. Moreover, while TMFs have species with a narrow range distribution, numerous species are also widely distributed across the neotropics [27]. It is uncertain whether widely distributed TMF species have distinct phenology patterns across the neotropics or whether they have variable patterns but respond similarly to environmental drivers across their distribution ranges. Understanding how flowering patterns of species found in TMFs vary across different environmental conditions is critical for understanding the ecology of these forests. Also, this research opens the door for expanding analyses of seed ecology and vulnerability to climate change for species across TMF.

We assembled a large dataset of herbarium records corresponding to tree species found in northern Andes TMF, across their distribution range in the wide neotropics, which encompasses several climatic regions, to answer the following questions: (i) Is it possible to derive phenological status information from herbarium record labels and build phenological calendars for many tropical tree species? (ii) Is there some degree of flowering synchrony for species within specific climatic regions in the neotropics? (iii) Do species from TMF distributed across different climatic regions have variable or consistent flowering patterns across regions?

## 2. Methods

### (a) Species selection and retrieval of herbarium records

We used data on tree inventories from 16 permanent plots from the ‘Pichincha long-term forest dynamics and carbon monitoring transect’ [28] to obtain a list of species from tropical mountain forests on the northwestern slope of the Ecuadorian Andes as a reference forest to look for herbarium specimens across the neotropics. The initial flora list included 516 unique taxa that included species unequivocally identified to the level of subspecies, species and genus, and 82 taxa with ambiguous identification at the species level (*conferatur* or *affinis*). Species names were curated to eliminate duplicates and entries only identified at the genus level. The final species list included 444 species from 80 families (see electronic supplementary material, S1 and S2). All species names were validated based on the Checklist of the Vascular Plants of the Americas [29]. For each species, we retrieved synonyms from the Tropicos database (https://www.tropicos.org/home, accessed 30 June 2022) using the taxize R package [30]. The final list of 2908 entries of the original species names and their synonyms was used to search herbarium specimens.

We retrieved herbarium specimen records across the neotropics from the Global Biodiversity Information Facility (GBIF) database [31] via the GBIF API (https://api.gbif.org/v1/), and the Herbario Nacional del Ecuador (QCNE) database—Instituto Nacional de Biodiversidad (INABIO, https://bndb.sisbioecuador.bio/bndb/collections). The initial GBIF dataset contained 54 146 records, while the QCNE dataset comprised 10 881 records. Both datasets were curated to remove duplicates, records of subspecies and varieties that lacked species-level synonyms in Tropicos, and records missing information from specimen labels (e.g. field notes). The final dataset of herbarium specimens consisted of 47 939 unique records corresponding to 427 species from 80 families distributed across the neotropics (electronic supplementary material, S2). The dataset spans the years 1821 to 2022, with most records (89.5%) collected from 1980 onwards (Figure S3 A). For complete details of the search parameters, data filtering and cleaning procedures, refer to electronic supplementary material, S3.1.

### (b) Machine learning approaches to determine phenological status

We used Natural Language Processing, a machine learning algorithm, to determine the phenological status of each specimen record based on the information in the field notes. First, we created a training and evaluation dataset of 1913 specimen records by selecting records from the main dataset through stratified sampling. The training and validation dataset was used to compare the performance of different machine learning models and select the model with the highest predictive accuracy metrics.

Information from field notes was cleaned and converted to a numerical matrix using the ‘bag of words’ model. The model estimates the number of times a word appears in a text. We evaluated three approaches to predict from transformed field notes data whether a specimen was flowering. We estimated the performance of the models using fivefold cross-validation with five metrics. For full details of the machine learning model methods, see electronic supplementary material, S3.2.

Once we determined the best performance model, we retrained it on the entire training and evaluation dataset. This model was used to predict flowering for all records in the final dataset (*n* = 47 939). We followed the same cleaning procedure for the whole dataset as that described above applied to the training and evaluation dataset. For all subsequent analyses, we considered only specimen records predicted to be flowering (*n* = 14 938).

### (c) Construction of phenological calendars

To construct phenological calendars, we utilized predictions derived from our dataset of flowering records and circular statistics. Circular analysis methods are particularly well suited for describing the periodicity of flowering events in tropical climates, where seasonality is weak or absent [17]. For example, if the onset of flowering of two species is typically a month apart, but one is in December and the other in January, non-circular statistics would inaccurately interpret their flowering as 11 months apart within the same year. In contrast, circular statistics correctly identify these events as temporally close. To construct the phenological calendars, flowering dates were mapped onto a circular scale ranging from 0° to 360°, where 0 represents 1 January and each month corresponds to 30°. A maximum likelihood estimation (MLE) approach [32] was then employed to assess whether flowering peaks occur at specific times of the year. The MLE approach compares 10 models, which represent distinct distribution patterns: (i) one uniform distribution, indicating continuous flowering throughout the year; (ii) three unimodal distributions, representing a single flowering peak; and (iii) six bimodal distributions, representing two flowering peaks annually.

The MLE approach estimates the mean direction (mean flowering date) for each model distribution, as well as a concentration parameter (*k*) with higher values corresponding to narrower peaks, and proportional size parameter (*λ*), indicating the proportion of observations falling within the first peak. The model uses the Akaike information criterion to identify which model best fits the data distribution. To test the consistency of the models, we conducted 100 runs. If more than 75 model runs converged on the same model distribution, we assumed a consistent flowering pattern. All analyses were performed using the R package CircMLE [32] and the *circ_mle* function with standard parameters (see package documentation: https://github.com/cran/CircMLE). Circular figures were generated using the *plot_circMLE* function, which incorporated both observed and fitted data. In cases where the MLE approach produced inconsistent results (<75 model runs converged), we created circular figures using the results of the most prevalent models (typically two or three) and visually inspected these figures to select the most suitable model.

### (d) Assessing flowering synchronicity within climatic regions

**Figure 1.**
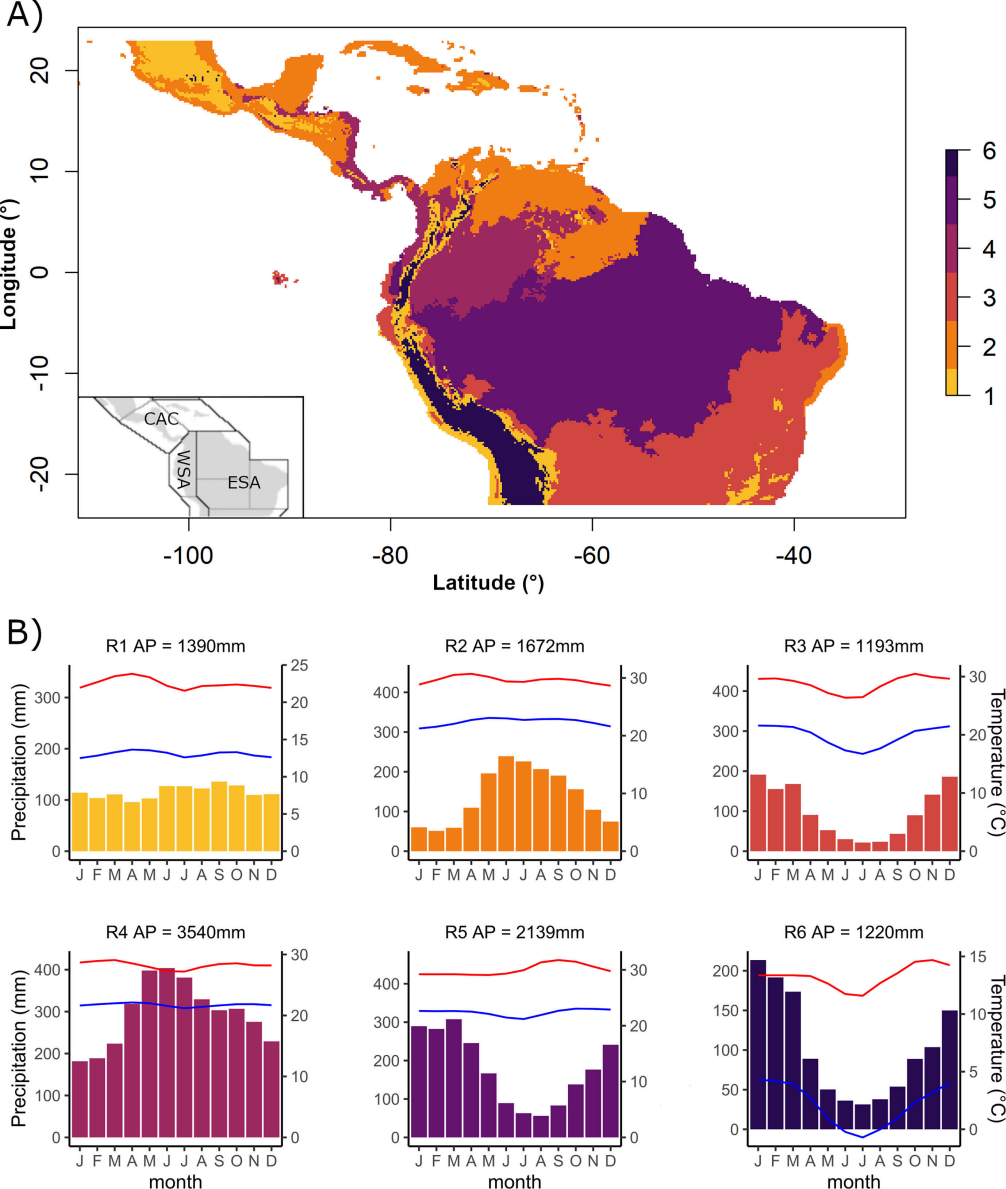
Geographical distribution of six climatic regions (R1 to R6) in the neotropics (A); the inner box represents the zones considered in the study: CAC, Central America and the Caribbean; WSA, west South America; ESA, east South America. Each climatic region has a distinctive seasonality (B) for precipitation (bars) and average temperature along the year (minimum temperature, blue lines; maximum temperature, red lines); AP, total annual precipitation.

We observed a wide distribution of occurrence records for the studied species across the neotropics, encompassing areas surrounding mountain ranges in the Andes and lowland forests in the Amazon and Central America. To assess flowering synchronicity, we defined distinct climatic regions within the range of specimen occurrences to account for the potential impacts of climate seasonality and light availability on phenological processes. We applied Zalamea *et al.*’s method [15] to delineate these climatic regions, using temperature and precipitation seasonality (see electronic supplementary material, S4.1). This approach enabled us to identify six distinct clusters that stratified the data into distinct climatic regions corresponding to well known biogeographical regions across the neotropics (figure 1).

Region 1 and Region 6 correspond largely to tropical montane forests along a broad latitudinal range. Region 1 includes mountainous areas in Central America, the Andean Piedmont and the Mantiqueira Mountains in Brazil, while Region 6 represents high-elevation areas such as the northern Andes’ paramos and the southern Andes’ puna (figure 1). The remaining four regions represent lowland ecosystems: Region 2 encompasses the Atlantic forests of northeastern Brazil, subhumid and dry forests of Central America, and xeric shrublands and the Llanos in Colombia and Venezuela. Region 3 corresponds to the Cerrado and Caatinga forests, extending between 5°S and 23°S. Region 4 includes moist areas along the Atlantic coasts of Costa Rica and Panama, the Chocó-Darien region and northwestern Amazonia, along the Napo, Caquetá and Río Negro rivers. Region 5 contains the moist Amazon Basin in Brazil and southwestern Peru, spanning between 5°N and 15°S. The distribution of occurrence records and species was uneven across these regions.

To evaluate flowering synchronicity within each region, we constructed phenological calendars by pooling species with at least 20 flowering records within each defined climatic region. Using the pooled data, we generated circular figures for each region (detailed model results provided in Table S4-B). Regions 1 and 2 covered a broad latitudinal gradient across Central and South America, whereas Regions 4 and 5 spanned a wide longitudinal range from east to west South America (figure 1). To account for this heterogeneity, we stratified these regions using two different and separate approaches: (i) we divided the neotropics into five latitudinal bands of 10° each, extending from 25°N to 25°S; (ii) following the Intergovernmental Panel on Climate Change (IPCC) climate reference subcontinental zones [33], we divided the neotropics into three broad climatic systems: Central America and the Caribbean (CAC), eastern South America (ESA) and western South America (WSA; see electronic supplementary material, S4.2). Subsequently, we constructed and assessed flowering calendars within these latitudinal bands or zones at each region. We found that IPCC zones partially overlapped with latitudinal bands within the regions. Therefore, our results focus on the stratification of regions and IPCC zones for both regional- and species-level calendars.

We also assessed flowering synchronicity by analysing the flowering patterns of individual species within each region and IPCC zone. Phenological calendars were created for species with at least 20 flowering records by region and IPCC zone to minimize confounding patterns due to mixing data from different geographical areas (see above), which can be particularly problematic for species with small numbers of records. By selecting flowering records based on regions and IPCC zones, we obtained 161 unique combinations (see electronic supplementary material, S5, Table S5-A), totalling 9810 flowering records from 86 species across six regions and three IPCC zones (CAC, ESA and WSA). We plotted phenological calendars for each species, including flowering dates determined by circular statistics. Where possible, environmental drivers such as seasonal drought and irradiance were included in the figures (Figure S5-B to S5-K in electronic supplementary materials S5).

### (e) Intraspecific variation in flowering patterns across regions

To assess whether species exhibited variable or fixed flowering patterns across regions, we selected species occurring in at least two regions and compared their phenological calendars. Environmental drivers, such as seasonal drought and (when possible) irradiance, were included in the figures (Figure S5-B to S5-K in electronic supplementary materials S5) to visually assess the possible coincidence of flowering peaks with peaks in irradiance or drought. We included precipitation seasonality for the corresponding region–zone combination in the flowering calendars. For seasonal irradiance, we searched for studies that estimated solar energy resources in the regions of our study. We found one study that presented seasonal calendars of global solar irradiance in northeast Brazil derived from measured data [34], and another study of global horizontal irradiance in Ecuador [35] from remote sensing data. We used the data from Brazil [34] to approximate seasonal irradiance in Regions 2–ESA, 3–ESA and 5–ESA. The study from Ecuador [35] was used to approximate seasonal irradiance in Regions 1–WSA and 4–WSA, where most records were concentrated near the Equator. For the other regions, we did not find regional studies to approximate seasonal irradiance; therefore, this information is not included in the flowering calendars. We assessed whether species across different regions and zones maintained consistent flowering patterns (uniform, unimodal and bimodal), whether they exhibited variation in flowering dates (patterns and dates were derived from circular models) and whether flowering peaks coincided with periods of seasonal drought or irradiance peaks (visual assessment, Figure S5-B to S5-K in electronic supplementary materials S5).

## 3. Results

### (a) Data coverage and predicting flowering with machine learning models

Out of the three classifiers evaluated with the training and evaluation dataset, random forest model (RFM) achieved the best performance metrics using labels of the specimen vouchers, with values greater than 90% on most of the goodness of fit metrics and low variability among model repeats (see electronic supplementary material, S3.2). When an RFM was applied to the entire dataset, it assigned 14 938 records as flowering and 33 001 as non-flowering (table 1).

**Table 1.** Data coverage of families, species and flowering status by number of records in the dataset after cleaning.

no. records^a^	no. families	no. species	no. occurrence records	no. catalogued as flowering	no. families flowering	no. species flowering
>500 (500−4342)	20	29	26 790	8047	3	3
100–500	31	53	11 498	3183	19	27
50–100	33	66	4758	1874	18	33
20−50	49	112	3471	1268	35	67
<20 (1–20)	48	167	1422	566	64	297^b^
Totals		427	47 939	14 938		427

^a^ Specimen records across the neotropics.

^b^ This number includes 41 species for which there were no flowering records.

Many species from TMFs used as a reference had a wide geographical distribution across the neotropics, with over 100 species present in at least five of the six climatic regions assessed. However, the distribution of both records and species was highly uneven. Only 7% of the species from herbaria had more than 500 occurrence records in the dataset, while 39% had fewer than 20 records (table 1). When considering only flowering specimens, fewer than 1% of species had more than 500 records, and the majority (69%) had fewer than 20 records. Species with more than 100 occurrence records were predominantly found at lower elevations (<1000 m) and were distributed across the entire latitudinal range of the study area (Figure S3-B in Sumpplementary material S3.1). In contrast, species with fewer than 100 records were more commonly found at higher elevations (>1000 m) and closer to the Equator.

Regarding the distribution of specimen records across the regions, Regions 4 and 1 accounted for 51−56% of the 90 species with at least 20 flowering records per region and 17.3−25.7% of all flowering records, with most species and records concentrated in the WSA zone (electronic supplementary material S4, table S4-A, S4-E and S4-G). Regions 5 and 3 accounted for 19−31% of the species and 10.5−34.2% of the flowering records, with most records concentrated in the ESA zone. Regions 6 and 2 had the lowest species representation (10−13%) and the fewest flowering records (3.7−8.6%), with most species and records concentrated in the ESA zone for Region 2 and the WSA zone for Region 6.

### (b) Flowering synchronicity within regions

**Figure 2.**
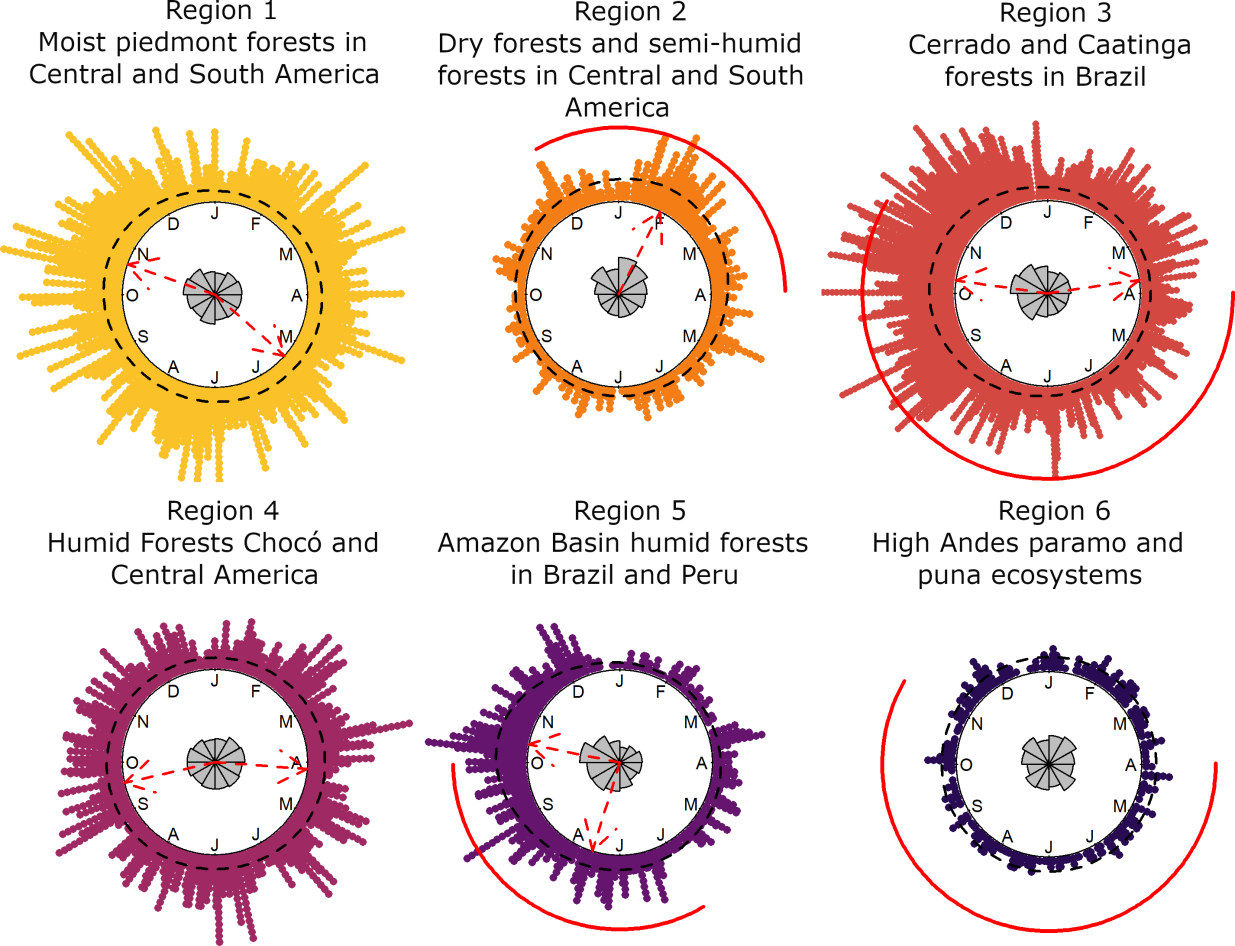
Circular plots of flowering patterns per region. Dots represent individual flowering records across years and grey bars show the cumulative number of records per month. Red dashed arrows indicate the mean direction of the modelled distribution (for bimodal distribution, each peak has its mean). The dashed black line represents the density of the distribution, while the solid red line marks the dry months (monthly precipitation <100 mm). For each climatic region, the corresponding biogeographical zones are included as a reference.

When pooling data of all species with at least 20 flowering records within a region, MLE models revealed continuous year-round flowering in five of the six regions, with weakly defined peaks characterized by a low concentration parameter (*k*; figure 2). The low flowering synchronization within regions partly reflects the aggregation of records spanning different latitudes and IPCC zones. Additionally, the weak coordination of flowering within regions and zones may be attributed to limited synchronicity among species-specific flowering patterns.

In mid-elevation TMFs of Region 1, flowering occurred throughout the year, with two weak peaks in May and October (figure 2). These peaks were primarily driven by records from WSA, which constituted 60.4% of the records and peaked in April and October, while in ESA (with 35.3% of the records), flowering peaked in October (electronic supplementary material, table S4-F and figure S4-F). Latitudinal differences within Region 1 further contributed to weak flowering peaks, with low synchronicity near the Equator and opposing flowering peak periods in the northernmost versus the southernmost latitudinal bands (electronic supplementary material, figure S4-B and S4-C). In Region 6, which encompasses high-elevation TMFs, nearly all records were from WSA, and flowering remained uniformly distributed throughout the year. Region 4 exhibited a bimodal flowering pattern with peaks in April and September, driven by WSA records, which accounted for 87% of the data. Regions 3 and 5 also exhibited bimodal flowering patterns, predominantly influenced by ESA records (>94% of the data). In Region 3, a primary flowering peak occurred in October, with a secondary peak in March–April, driven by 64% of records being from ESA. Region 5 had a main flowering peak in October and a secondary peak in July. Finally, Region 2, with the fewest records and species, displayed a single flowering peak in February. This region was dominated by records from ESA, accounting for 83% of the data.

We analysed flowering patterns for 86 species across the six regions (encompassing 161 species within region–zone combinations) to assess species-level flowering synchrony. There was substantial variability in flowering patterns among species within regions. In TMF Region 1–WSA, its 40 species exhibited unimodal, bimodal and uniform flowering patterns in nearly equal proportions. Ten out of the 13 bimodal species in Region 1–WSA peaked around March and October, coinciding with periods of high seasonal irradiance (electronic supplementary material, figure S5-B). Flowering of unimodal species did not exhibit clear associations with precipitation or irradiance.

In TMF Region 6, all species displayed uniform flowering throughout the year. In Region 4–WSA, 43% of the 42 species followed bimodal patterns, with 12 of these 18 bimodal species peaking around March and September, corresponding to periods of high irradiance. The remaining bimodal species showed flowering peaks in June and December. Most unimodal species (7 out of 11) peaked around June.

In Regions 3–ESA and 5–ESA, most species were unimodal (>60%) or bimodal. In Region 3–ESA, 9 of the 10 bimodal species peaked in March–April and October–November, whereas 9 out of 17 unimodal species peaked between October and December. In Region 3–ESA, irradiance peaked in September and November, aligning with the end of the dry season. In Region 5–ESA, 7 out of 10 unimodal species flowered in July or November–December, and most bimodal species (*n* = 4) had peaks from May to July and again from October to December. These peaks partially overlapped with the June–September drought period, though they did not align with the high irradiance months of February and October. In Region 2–ESA, only 10 species were recorded, which exhibited bimodal or unimodal flowering patterns. Seven of these species had at least one flowering peak between February and March (Figure S5-B to S5-K in electronic supplementary materials S5).

### (c) Intraspecific variation in flowering patterns

Across the six climatic regions, 47 species were exclusive to a single region, while 39 species were distributed across two to five regions. Nineteen species occurred in different regions in both ESA and WSA, eight species were restricted to ESA across two to four regions, and 12 species were confined to WSA across two regions. Among the 39 species found in multiple regions, only six exhibited similar flowering patterns and timing across regions and zones, even when they aligned with different environmental drivers. The remaining species exhibited variable flowering patterns, flowering dates or flowering peaks that coincided with different environmental drivers across regions (see electronic supplementary material S5, tables S5-B to S5-D).

**Figure 3.**
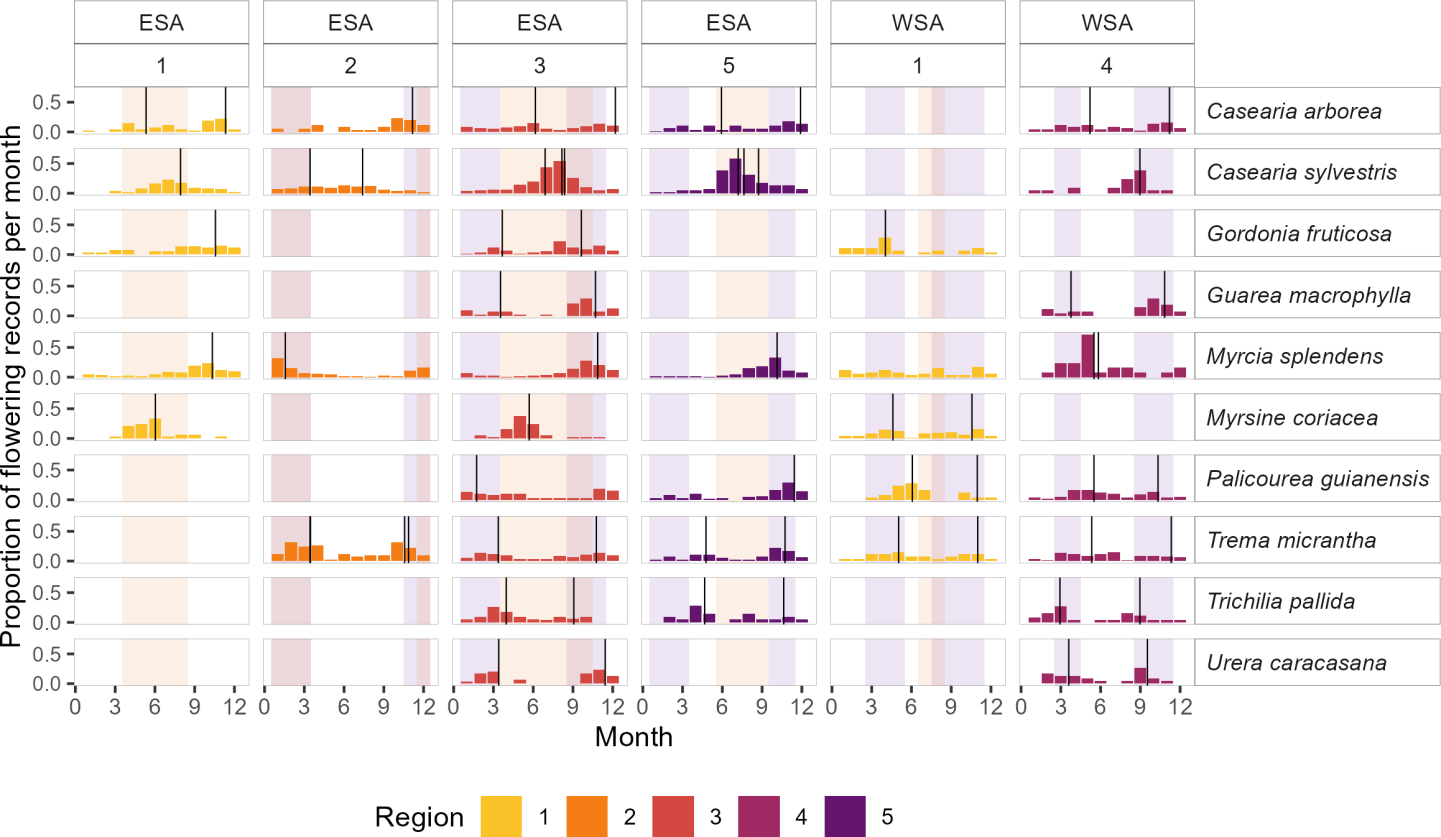
Flowering calendars for species occurring in more than two regions and at least two zones. To facilitate comparison among species with different numbers of flowering records, we present the proportion of flowering records for each species, normalized by the maximum number of flowering records in any region–zone combination for that species. Vertical lines indicate flowering peaks estimated through circular statistics. Shaded areas represent months that coincide with seasonal drought (light orange) and peaks in irradiance (light violet).

Of the 19 species present in both ESA and WSA, six exhibited consistent flowering patterns across regions and zones. For instance, *Casearia arborea* showed no coincidence with rainfall or irradiance patterns, while *Casearia sylvestris* aligned with seasonal drought. *Trema micrantha*, *Trichilia pallida*, *Guarea macrophylla* and *Urera caracasana* displayed bimodal flowering near periods of high irradiance (figure 3). In contrast, *Gordonia fruticosa*, *Myrsine coriacea* and *Myrcia splendens* had similar flowering dates in ESA but varied in WSA. *Gordonia fruticosa* and *Myrsine coriacea* showed variable flowering patterns and flowered near irradiance peaks, while *Myrcia splendens* was mostly unimodal and coincided with irradiance in ESA, though not in WSA. *Palicourea guianensis* showed consistent flowering patterns and dates only within WSA, with at least one flowering peak during periods of high irradiance. The remaining nine species exhibited variable flowering patterns, dates or variable coincidence with potential drivers across regions (see Figure S5-L in electronic supplementary material, S5).

**Figure 4.**
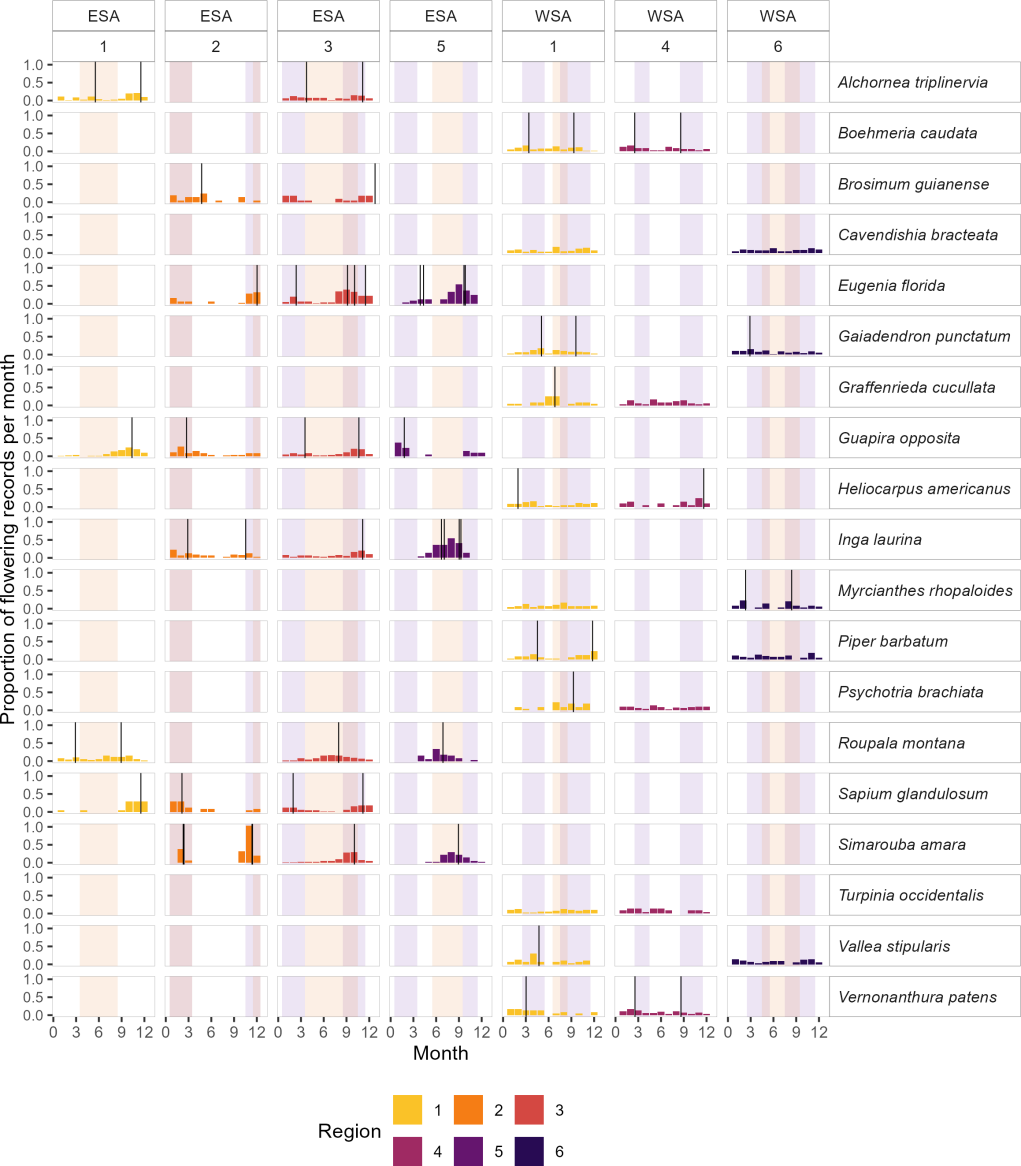
Flowering calendars for species from two regions within eastern South America (ESA) and western South America (WSA). Bars, lines and colours are consistent with those in figure 3.

Among the eight species found in multiple regions within ESA, four species, *Guapira opposita*, *Sapium glandulosum*, *Simarouba amara* and *Alchornea triplinervia*, exhibited unimodal or bimodal flowering, with peaks in February–April and October–December, coinciding with at least one period of high irradiance (figure 4). The remaining species exhibited variable flowering patterns and dates within ESA. In WSA, only 3 of the 12 species recorded across multiple regions showed consistent flowering patterns: *Boehmeria caudata* with bimodal flowering, while *Cavendishia bracteata* and *Turpinia occidentalis* displayed uniform flowering patterns (figure 4). Of the 26 species with variable flowering patterns and dates, only seven seemed to have flowering peaks close to irradiance peaks (*Acalypha diversifolia*, *Eugenia florida*, *Guarea kunthiana*, *Nectandra membranacea* and *Vernonanthura patens*) or periods with drought (*Ossaea micrantha* and *Roupala montana*).

## 4. Discussion

### (a) Using herbarium records to build phenological calendars

We constructed phenological calendars for 86 out of 444 TMF species from the Ecuadorian northern Andes across their geographical distribution in the neotropics, aiming to identify potential phenological patterns across their occurrence range. Our approach leverages years of specimen collection and uses novel analytical tools, such as machine learning algorithms and circular statistics, to gain insights into the phenology of these understudied montane forest species.

Using herbarium records for phenological studies requires determining the phenophase of interest. Significant efforts have been made to develop standardized scoring methods to categorize specimens into phenological phases [36] and to assign phenophases through visual inspection by trained individuals or image analysis software [9]. However, these approaches are limited by the time and expertise required to apply them to large datasets. This study demonstrates that machine learning methods can successfully process extensive datasets containing qualitative information, such as herbarium specimen labels, to categorize the flowering phenology of TMF species. In our study, the algorithms were trained to assign specimens into simple flowering/not flowering classes. Depending on the level of detail in the label descriptions, it may be possible to train models to obtain other phenological information, such as ‘open flowers’, ‘flower buds’ or ‘senesced flowers’. However, capturing more detailed phenological traits such as the number of flowers or fruits, the proportion of immature versus fully open flowers [36] or the specific onset of phenological events, like the first flowering date, would require different methods.

We estimated the mean flowering dates or peak of flowering times using circular statistical approaches. The circular statistical approach applied in this study has mainly been used with animal orientation data [32], where sample sizes as small as 20 observations are sufficient to detect unimodal distributions, but the *k*-parameter should be greater than 1. The *k*-value is a concentration parameter that specifies data distribution, where high *k*-values indicate narrow, more pronounced peaks. As the sample size increases to around 100 observations, the *k*-value can decrease to as low as 0.4 while still allowing the detection of unimodal or bimodal distributions. This suggests that larger sample sizes enable the discernment of even subtle peaks in distributions with broad dispersion. In our study, models built for entire regions, latitudinal bands and zones within regions had large sample sizes (*n* > 100) and low k-values (electronic supplementary material S5, figure S5-A). For the individual species models (161 species within region and zone combinations), 70% had fewer than 50 records. Only seven species had a *k*-value lower than 1 among those with fewer than 50 records, and only one species had a *k*-value lower than 0.4 among those with more than 50 records. Therefore, despite limited sample size and wide geographical dispersion of some species, we could reliably estimate flowering periods for most of the species studied.

Our results also showed that *k*-values tended to decrease (indicating less pronounced peaks with larger dispersion) with increasing sample size, reflecting the highly variable nature of flowering events over extended time and geographical scales. We used flowering records spanning a considerable period, expecting that flowering peaks would reflect stable long-term patterns; nevertheless, it is important to recognize potential confounding factors. Extreme climatic events such as El Niño, microclimate variations or supra-annual and multiple peak flowering patterns [19] could contribute to increased variability in flowering dates. Another relevant aspect is the potential influence of climate change on shifting flowering periods [12,13]. However, since 90% of herbarium records were collected after 1980, it is likely that the observed flowering patterns predominantly reflect baseline climatic conditions.

### (b) Synchronicity of flowering patterns of tropical montane forest species in the neotropics

Partitioning the neotropics into six regions characterized by similar temperature and precipitation regimes effectively separated TMF (Regions 1 and 6) from lowland forests. The models detected weak flowering peaks with a wide dispersion in five of six regions. The considerable dispersion of flowering events throughout the year indicates limited interannual synchronicity in flowering patterns within regions. This intra-regional dispersion of flowering was partly attributed to latitudinal differences, with weaker flowering peaks (low *k*-values) closer to the Equator in Region 1. However, it was more strongly linked to variation in the flowering patterns of individual species. Flowering patterns varied considerably among species within each region. Bimodal species exhibited more synchronous flowering than unimodal species, with most bimodal species in Regions 1, 3 and 4 peaking in March–April and September–October, coinciding with periods of higher irradiance and the end of the dry season in Region 3. In contrast, bimodal species in Region 5 exhibited peaks in June–July and November–December, with at least one peak aligning with the seasonal drought, though no clear environmental driver was identified for the second peak. Unimodal species generally exhibited little synchronization in flowering across all regions. The variable flowering patterns of unimodal species, along with the continuous flowering of uniform species (particularly common in Regions 1, 4 and 6), contributed to the limited annual synchronicity observed within regions.

Numerous studies on tropical lowland and mountain forests have shown that environmental drivers, such as rainfall and irradiance, influence phenology [21–23]. Our findings suggest that irradiance may be a critical driver in shaping flowering patterns, particularly for bimodal species. For unimodal species, flowering patterns were more complex and variable, indicating that additional environmental or biological drivers may also play a role. Although we cannot fully characterize entire communities since the selected species represent only a fraction of the forest communities in the assessed regions, our results align with previous research suggesting a great diversity of flowering periods in tropical regions [20,23]. Variable flowering patterns have been linked to a fitness advantage, which reduces interspecific competition during reproduction, thereby promoting species coexistence in tropical localities with little or no seasonality [18].

### (c) Intraspecific variation in flowering patterns

Among the 39 species occurring in multiple regions, only six exhibited similar flowering patterns and dates across regions and zones, even when they aligned with different environmental drivers. Seven species showed some consistency in flowering patterns or dates within at least one zone, while the remaining 26 were highly variable. This variability in phenological responses at the species level may derive from phenotypic plasticity, indicating an adaptative capacity to adjust to changing climates or other environmental factors, or from genetic variability. While genetic variability can facilitate evolutionary adaptation to the local climates, it may also become problematic if there is a mismatch between environmental drivers and genes that could potentially lead to population decline [37].

Our results suggest that the flowering of the species present in more than one region is mainly plastic, with 85% of species exhibiting variable flowering patterns or dates across regions. This plasticity manifests as intraspecific differences in flowering patterns (i.e. bimodal, unimodal or uniform), flowering dates and possible links to environmental drivers, highlighting the complex interplay between species’ reproductive physiology and regional climates.

Regarding environmental drivers, 14 out of 39 species had one or two flowering peaks aligned with periods of high irradiance; only four species aligned with drought periods. For the remaining species, the environmental drivers varied across regions. These findings emphasize the role of irradiance as a flowering trigger in the tropics [21,22,24,25], and open research questions about the importance of reproductive plasticity as an adaptation to reduce reproductive competition [18], particularly under climate change.

Nevertheless, it is important to bear in mind that our findings about flowering drivers are mainly qualitative owing to methodological constraints and data scarcity. We used climatic data derived from interpolations of weather stations and remote sensing data [38] scaled to a 20 km × 20 km grid (Methods), and estimates of irradiance seasonality from secondary sources, also scaled at national and regional levels [34,35]. A more detailed exploration of climatic drivers affecting phenology would require finer-scale climatic data linked to each species’ occurrence record by date and location [39,40]. Additionally, such analyses will depend on data availability to apply new statistical methods [41]. Nevertheless, these methods require large sample sizes (e.g. *n* = 200) for detecting the effects of a single continuous predictor [41], which may pose challenges in data-constrained settings. Furthermore, at this time, these methods can only be applied with normal circular data, which is defined as normal unimodal distributions projected on a circular scale.

### (d) Filling information gaps

Herbarium records from our northern Ecuadorian Andes species list revealed a wide neotropical distribution, reflecting the complex biogeographical origins of Andean Forest species [27]. Most records were concentrated in Regions 1, 4 and 6, closer to the Equator. Central America had the fewest records overlapping with our sampled forest.

Despite our large dataset, many TMF tree species (approx. 300 species) lack sufficient data. Herbarium collections often oversample rare species owing to collection strategies that avoid duplicates and prioritize rare finds [42], leading to poor documentation of many tropical species.

When comparing the coverage of herbarium records with the abundance of the selected species from the ‘Pichincha long-term forest dynamics and carbon monitoring transect’ [28], we observed that herbarium records were particularly useful for gaining knowledge of rare species in TMFs. Of the 86 species for which we could build phenological calendars, 73% corresponded to species that had only one or two individuals across the TMFs census used to populate the species list of this study. This list included both common species in herbarium records (i.e. very abundant in lowland forests, such as *Myrcia splendens*, *Trema micrantha*, *Inga laurina*, *Casearia sylvestris* and *Guapira opposite*) as well as rare species in the overall sample.

However, there are still many species for which phenological information is lacking. For example, of the 20 most abundant species in the reference TMFs, we were only able to build calendars for six: *Axinaea macrophylla*, *Meriania tomentosa*, *Miconia theaezans*, *Ocotea insularis*, *Palicourea amethystina* and *Weinmannia pinnata*. Our analytical workflow can help guide targeted data collection efforts to address these gaps. Future strategies could focus on studies prioritizing abundant species in TMFs, which are under-represented in herbarium collections and previous studies.

Finally, we could not use information from non-flowering records, which accounted for 60% of the total records for the selected species, because flowering calendars constructed with circular statistics and other methods rely only on flowering data [14,15,17]. Exploring alternative approaches incorporating both flowering and non-flowering data could offer valuable insights. However, these methods would need to address challenges associated with non-flowering records (e.g. a specimen not in flower might not accurately reflect the true phenological status of the plant, particularly when collected from young plants or non-flowering plant sections) .

In conclusion, this is the first attempt to provide insights into the phenology of numerous tree species from TMFs using open-source data and novel analytical tools that leverage decades of herbarium collection. We created phenological calendars for six regions and 86 species within region–zone combinations across the neotropics, revealing a great diversity of flowering patterns among regions and within species across different regions. Notably, we observed some degree of synchronic flowering within regions, particularly among bimodal and some unimodal species, which appears to be related to irradiance. Our results significantly increase the current understanding of the reproductive biology of various little-known TMF tree species. Future research efforts should prioritize the targeted collection of phenological data for critical species (i.e. the abundant species from TMF that have limited coverage in herbaria or other phenological studies) for flowering and, more critically, for fruiting phenology for which information is even scarcer. Additionally, a deeper exploration of environmental drivers and the role of phenotypic plasticity in flowering as an adaptive mechanism will further clarify the fitness benefits and resilience of TMF species in changing climates.

## Data Availability

All scripts and datasets for running machine learning models and circular analysis to construct phenological calendars and paper figures are publicly accessible via these links: data [43]; scripts: [44]. Supplementary material is available online [45].
